# A Comparison of Methods for Testing and Implementing Community Health Interventions in Childhood: A Realist Review †

**DOI:** 10.3390/children12121605

**Published:** 2025-11-25

**Authors:** Lubna Anis, Karen M. Benzies, Carol Ewashen, Martha Hart, Nicole Letourneau

**Affiliations:** 1 Owerko Centre, Alberta Children’s Hospital Research Institute, Department of Pediatrics, Cumming School of Medicine, University of Calgary, Calgary, AB T2N 1N4, Canada; mhart@ucalgary.ca (M.H.); nicole.letourneau@ucalgary.ca (N.L.); 2 Faculty of Nursing, University of Calgary, Calgary, AB T2N 1N4, Canada; benzies@ucalgary.ca (K.M.B.);

**Keywords:** interventions, randomized control trials, rapid-cycling trials, fast-iteration research design, fast-fail trials, vulnerable children and families

## Abstract

**Highlights:**

**What are the main findings?**

- Traditional randomized controlled trials (RCTs), although strong in internal validity, are often too rigid and slow, limiting their suitability for evaluating complex community-based child health interventions.
- Innovative and accelerated evaluation methods show greater promise for real-world application, with the IDEAS (Innovate, Develop, Evaluate, Adapt, Scale) framework emerging as the most integrative, theory-driven, and context-sensitive approach for evaluating early childhood interventions.

**What are the implications of the main findings?**

- Child health research and practice are well positioned to adopt adaptive, context-responsive approaches such as the IDEAS method to support faster learning, mid-course adaptations, and greater relevance across diverse community settings.
- Broader use of IDEAS can enhance rapid implementation and improve equitable, scalable outcomes for vulnerable children and families.

**Abstract:**

**Background:** Innovative methods to test healthcare interventions have recently emerged to help provide more targeted, effective, and scalable interventions. Given the importance of the early years for children’s development, improved interventions for vulnerable children and families have become public health imperatives. Traditional randomized control trials (RCTs), considered the gold standards, have serious limitations due to high costs, time demands, and issues with the generalizability of the results. Indeed, new accelerated methods are being considered to improve the efficiency of RCTs. Thus, we compared innovative methods with RCTs in their ability to test and implement interventions. We also provided recommendations for best practices in the child-health research. **Methods:** A realist review was undertaken to identify and make recommendations on what works for whom and under what circumstances. This realist meta-review was conducted as an umbrella review of reviews, supplemented by a synthesis of the targeted grey-literature, to report both peer-reviewed and practice-based evidence on evaluation methods for community child-health interventions. We searched electronic databases, including MEDLINE, PubMed, EMBASE, PsycINFO, CINAHL, and the grey literature, and provided references. We identified, selected, and appraised sources if they were (1) written in English, (2) answered our research question, (3) described/criticized a method for intervention evaluation, and (4) focused on community-based health interventions. **Results:** For our final analysis, out of 5167 identified documents, we selected those that criticized or reviewed RCTs (*n* = 13) and innovative methods (*n* = 31). Following Pawson’s recommendations, we developed an extraction tool to promote a consistent approach and assessed to what degree each method enabled evaluation, was theory driven, offered clear guidelines, provided clear methods or tools, fostered innovation, was fast and generalizable, worked for who and under what circumstances, and focused on children and child-related research. **Conclusions:** Innovative and accelerated methods offer promising alternatives to the traditional RCTs for evaluating community-based child health interventions. Among these, the Innovate, Develop, Evaluate, Adapt, and Scale (IDEAS) method emerged as the most integrative and context-sensitive approach to evaluate early interventions in a variety of settings. Other innovative methods were not well-developed, compromising the internal validity of studies focused on promoting children’s health in community settings. Graphical abstract synthesizes the phases of RCTs and contrasts them with IDEAS.

## 1. Introduction

First described in 1747 by James Lind, traditional randomized control trials (RCTs) have become the gold standard for evaluating the efficacy and effectiveness of interventions for various populations [1,2], including children in clinical (e.g., hospital) and community settings (e.g., agencies offering intervention for children at high psychosocial risk) [3,4,5]. The characteristics of manipulation, randomization, and control of RCTs reduce or eliminate many forms of bias that may undermine internal validity. As such, RCTs yield the most robust evidence for causal relationships necessary for implementing findings in clinical practice in many disciplines, including medicine, nursing, psychology, social work, and education [6]. Nonetheless, RCTs suffer from limitations such as high costs due to the necessity for large sample sizes, extensive staff training, continuous monitoring, and lengthy follow-up. RCTs are often criticized for selective outcome reporting (gaming primary outcomes), data dredging, the Table 1 fallacy (misinterpreting or misleading baseline comparisons), reliance on middle-class samples, and a lack of robust process or qualitative evaluations [7]. Echoing James Lind’s call to clear rubbish from treatment claims through fair tests, the James Lind Alliance now puts this into practice by convening priority setting partnerships of patients, carers, and clinicians to agree on the top ten research priorities that expose gaps between academic/funder agendas and real-world needs [8].

With respect to interventions for populations of ill children or children at risk for health and developmental problems, the time delay between testing interventions and mobilizing knowledge may be too slow for rapidly growing children [9,10,11]. In the age of precision population health, it is increasingly urgent to understand what works for whom to promote children’s health and development [12]. Further, popular movements such as the emergence of anti-vaccination and holistic health movements require fast and compelling evidence of the efficacy and safety of alternative treatment approaches [13]. It is essential to uncover newer, innovative, and faster methodologies to identify effective interventions to address emerging community healthcare challenges, especially related to children’s health. In this paper, we conducted a realist review to compare emerging methods for intervention evaluation and mobilization with each other and with traditional RCT methods, primarily related to the research on children’s health and development in the community. To begin, we will review the specifics of RCTs as our comparator.

### Randomized Controlled Trials

RCTs have prevailed over clinical judgment, case reports, and observational studies in healthcare everywhere, including in the study of interventions affecting child health and development [1,14,15]. Children’s evidence-based care may be guided by the systematic reviews and meta-analyses based on the results of RCTs [14,15]. In RCTs, participants are assigned to one of at least two comparison groups on a random schedule, of which one group must be a viable control group, where only one variable can be manipulated [1,6]. RCTs are comprised of five different phases, including the pre-clinical phase [2,6,16]. The pre-clinical phase involves identifying a clinical problem through literature searches, and exploring existing evidence in support of the possible advantage of the intervention being tested [6,17]. This phase also provides the theoretical groundwork for the intervention to be tested in the next phases of RCTs [16,18,19].

In Phase 1 of RCTs, specific intervention components are empirically tested for the first time in the real world to determine effectiveness, feasibility, and acceptability [16,20]. Phase 1 of RCTs typically includes feasibility [16,17,18,19,21,22,23] and pilot studies [17,18,22] conducted before the commencement of the future main RCTs to provide the foundational groundwork. In Phase 2 of RCTs, an intervention is initially tested in comparison with an alternative treatment to determine efficacy [24]. Phase 2 of RCTs typically has small sample sizes (e.g., 30–60), but is conducted to generate effect sizes to determine the efficacy of an intervention [6]. Phase 3 of RCTs typically includes double-blinded, large RCTs to test a fully developed intervention with a larger sample size (e.g., 100–3000) [24]. Phase 4 of RCTs is conducted to provide clear potential direction toward individual or public benefit by moving from assessing efficacy to sustaining in clinical practice [24,25]. In this phase, interventions can be tested for effectiveness with diverse groups of participants and replicated in other settings [6,26]. Also, the focus of Phase 4 is to monitor quality and fidelity of intervention delivery and its enactment. Twelve to eighteen years may be required for an intervention to be tested through all RCT phases [27,28]. Nonetheless, RCTs are employed to enhance reliability relating to the effects of the intervention due to reduction in confounding factors, selection bias and interpretation bias [29].

RCTs suffer from several limitations, such as problems with the generalizability of results, inflated costs associated with long-term follow-up, large sample sizes, and extensive staff training. Participants enrolled in RCTs must meet strict eligibility criteria and may not represent the population, which may affect the generalizability of results [29,30]. Due to high costs and participant attrition, it may be challenging to evaluate the longer-term effects of an intervention, and thus very rare harmful effects of the intervention may not be detected by RCTs [2,24,31]. Although researchers intend to test interventions on large sample sizes, this is not always possible, and the number of participants enrolled is always less than the populations affected by a specific illness [32]. Finally, RCTs prohibit researchers from making mid-course adaptations based on their learning during different phases of RCTs [27,33].

Furthermore, RCTs have classically been used to determine the efficacy of an intervention, or in other words, to determine whether it results in the desired outcome in a highly controlled, ideal environment [34]. In contrast, effectiveness trials are conducted to determine how well the intervention works in actual, real world conditions [34]. This is an important distinction because while efficacy indicates the potential of an intervention under optimal conditions, effectiveness tells us about the generalizability and practical importance of an intervention when implemented in a variety of day-to-day settings [35]. While RCTs have been instrumental in establishing causal relationships, their explanatory focus often overlooks how and why interventions work in real-world contexts, particularly those designed to promote children’s health in community settings. To address these gaps, realist and implementation research perspectives emphasize knowledge-mobilization approaches that explore mechanisms of change and contextual influences, helping to bridge not only the know–do but also the do–know gaps [12,36,37,38,39].

The traditional RCTs have long shaped evidence base in child health, yet they often underrepresent the contextual and social complexities of real-world interventions. This realist-informed review builds on a long tradition of pragmatic, practice-oriented inquiry from James Lind’s applied problem-solving for sailors to John Dewey’s philosophy of pragmatism and later frameworks of engaged scholarship and theory-in-practice [40,41], each emphasizing the generation of actionable knowledge from real-world settings. These perspectives, now echoed in contemporary knowledge mobilization and rapid/relational evaluation approaches [37,38,42,43], highlight the enduring importance of designing evaluation frameworks that close the persistent do–know gap by linking research, implementation, and practice.

New accelerated methods are increasingly employed to reduce costs and improve the efficiency of clinical trials. Examining the differences among innovative methods and contrasting them is critical to understanding the best approaches for testing interventions. Thus, this paper aims to compare intervention testing and implementation methods to provide recommendations for research to improve child health in community settings. For this review, RCTs were defined as studies which use randomization to assign participants to an experimental and a control group and the primary aim is to establish causality in a controlled environment (pre-clinical, Phase 1, Phase 2, Phase 3, and Phase 4). This operational definition was applied consistently to identify and compare methodological frameworks throughout the review.

## 2. Materials and Methods

A realist review method was employed, which involves integration and synthesis of a diverse selection of literature for making recommendations about what works for whom and under what circumstances, and specific to this review, for child health and development intervention research in community settings [37,38,43]. The rewards of realist review methods include the potential for more pragmatic conclusions compared to alternative approaches to systematic reviews [37]. Realist reviews support the utilization of high-quality evidence, but the evidence is judged based on relevance to the research question and whether a credible inference may be drawn [44]. This approach necessitates the inclusion of a variety of source materials based on the researchers’ judgment of the usefulness of the information. Potential sources include peer and non-peer-reviewed studies, internal program evaluations, and unpublished but publicly available research reports. Replicability is de-emphasized in favor of topic- and context-specific expertise and critical evaluation to guide the analysis of the literature [44]. Consistent with Pawson [37,38] and Wong [43], the present realist review [44] included five stages.

### 2.1. Stage 1: Identifying the Research Question

#### 2.1.1. Research Question

How do emerging and adaptive methods for intervention evaluation compare with traditional randomized controlled trials (RCTs) in their ability to evaluate and implement interventions to support children’s health and development in community settings?

#### 2.1.2. Aim and Objectives

This realist review aimed to assess how the traditional RCTs and newer adaptive designs generate timely, context-sensitive evidence to improve child health in community settings by (1) mapping their use, (2) comparing strengths and limitations for explaining what works, how, for whom, and under what circumstances, and (3) examining their interface with implementation research.

#### 2.1.3. Scoping and Rationale

We clarified the scope and refined the research question by conducting an exploratory review of the literature on intervention evaluation methods. This strategy enabled us to initially identify the names of new emerging methods and search terms, including rapid-cycling trial, fast-iteration research, and fast-fail trial, in combination with the term’s method/methodology and design. This scoping phase was intentionally realist in orientation, emphasizing theory-driven exploration rather than predefined hypotheses.

### 2.2. Stage 2: Underlying Theory and Mechanism

Rooted in scientific realism, a realist review is a theory-driven method of providing contextually relevant evidence [43]. Consistent with a realist-informed design [43] rather than a purist realist approach, we applied context–mechanism–outcome (CMO) principles to structure theory-driven inquiry and interpretive synthesis—without formal CMO mapping or mid-range theory testing—to understand how evaluation designs operate across contexts and for whom they are most applicable [44]. Aligned with the purpose of review, we used Hertzman’s perspective on how social environments shape human development as the guiding program theory to ask what works, for whom, why, and in which context [45,46]. Focusing on social determinants of early child development [45,46,47,48,49,50], Hertzman’s theory explores how the health and well-being of infants and children can be best supported in an era of rapid economic and technological change. Hertzman coined the term biological embedding for the first time to describe how early life experience change the human brain and behavioral processes [51].

Following on from Hertzman’s theory [45,46] that early life experiences are incorporated under the skin to shape the brain and lifelong health, our evaluation framework is intended to capture not only internal validity of intervention methods, but also their sensitivity to contextual and social determinants. In practice, this means that we assess methods based on their ability to: (1) incorporate a clear theory of change that maps how interventions affect developmental processes, (2) adapt to the dynamic interplay between the individual and environment, and (3) support iterative, context sensitive evaluations [48,51]. To elaborate, Hertzman explored the links between socioeconomic status and health and how environmental characteristics (e.g., social support) and early, typically community-based, interventions can develop innovative kinds of learning environments to nurture adaptation and intellectual growth [45,46,47,48,49,50,51].

### 2.3. Stage 3: Search Process and Selection of Documents

We searched electronic databases (MEDLINE, PubMed, EMBASE, PsycINFO, and CINAHL) and grey-literature sources between 2019 and 2022 to identify reviews that described or criticized evaluation methods for community-based interventions. We searched with MeSH term (including entry terms) using the or/and combination using the following terms: (1) for traditional RCTs, intervention, randomized control trial or randomized control trial or RCT, method or methodology, design, evaluation, critique, and review (2) for innovative clinical trials, new or innovative, rapid cycle, fast cycle, clinical trial, intervention, evaluation, fast iteration, method or methodology, fast fail, critique, adaptive, and design.

We did not include the term child development in our search with an expectation of finding articles that have been developed for evaluating the interventions for adults but can be adapted for intervention testing for children. The decision to leave out the term child development from the search strategy was intentional. The approach of the review was designed to identify a wide range of intervention evaluation methods developed and validated in adult populations, knowing that many of these methods can be used for pediatric research after some adjustments. In other words, the assumption was that if a method fits for the evaluation of interventions in adults, then the basic concepts underlying the method can be used for children with some changes. Employing this strategy ensured, the authors could consider a greater number of established methods than possible by limiting those methods focused on child development. Additionally, effectiveness-implementation hybrid designs [52] were excluded as they are methods that simultaneously assess intervention effectiveness and outcomes in implementation studies, rather than accelerated designs. We identified additional articles using ancestry searches of reference lists from retrieved papers.

Consistent with a review-of-reviews design, our primary sources were existing systematic, narrative, or scoping reviews that examined evaluation methodologies. We did not extract new data from primary studies; instead, individual studies were revisited in later sections only to illustrate specific methodological or contextual insights identified through the reviewed literature. Given the rapid innovation in this field, we searched for grey literature, relevant reports, clinical trials website, theses and dissertations, newspapers, government documents, conference proceedings, posters, and applications (or apps). We approached experts in the field to identify source documents of interest by purposive sampling (senior/corresponding authors of methodological papers and leaders of reputable organizations). We also searched institutional and organizational websites using our predefined terms and, through snowballing in our professional networks, identified additional grey literature (e.g., unindexed conference presentations and reports). We conducted individualized outreach to request methodological overviews, publicly available grey literature, and further contacts, and we tracked all communications in a contact log. To identify grey literature unavailable in journal databases, we used snowball searching, such as pursuing references of source documents using Google, Google Scholar, and DuckDuckGo search engines. We continued snowball searching from January 2019 and February 2022 to include all relevant literature.

In keeping with a realist-informed design, we developed inclusion and exclusion criteria to justify how the team decided to include and exclude data. We included sources if they were (1) written in English, (2) answered our research question, (3) described/criticized the method, and (4) focused on community-based interventions. We excluded sources if they were primary trial reports and focused on pharmacologic studies/drug trials.

**Figure 1:**
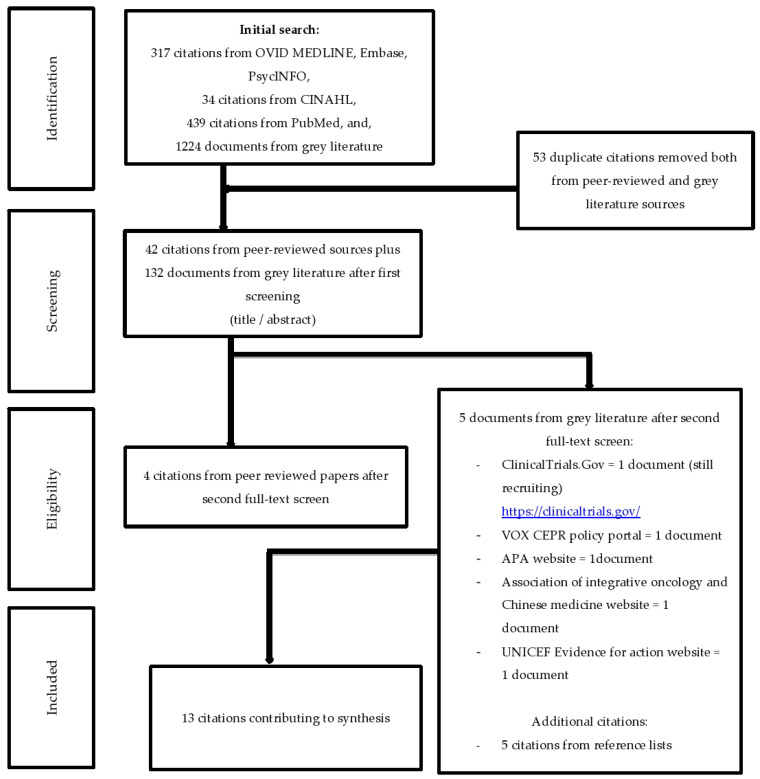
Study selection flow for the RCT review.

**Figure 2:**
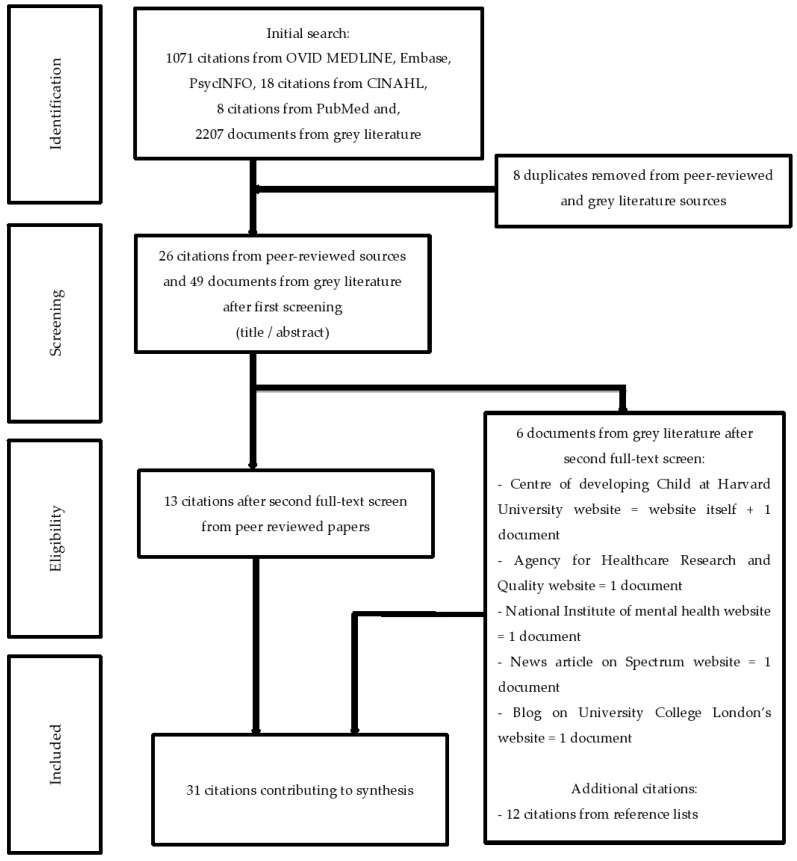
Study selection flow for the innovative/accelerated methods review.

Innovative methods were defined as novel approaches developed to capture the effectiveness of interventions while decreasing cost and time associated with RCTs. Such methods were recognized by their characteristics which include flexibility, quickness, and adaptability to context. Subcategories within innovative trial methods included approaches that enabled mid-course adaptation and context-oriented appraisal. This classification was carried out both on theoretical basis (for instance, Hertzman’s principles on the importance of context in child development) and empirical findings [45,46]. To make our data extraction protocol more specific and easier to follow, we identified specific documents to be included and excluded in the review. We only included those documents which were most relevant and most methodologically sound in the review. The process of selection began with a title and abstract screening, then a thorough full text analysis. In the full text phase, any paper that did not provide certain data was classified as not reported. This approach was first applied by the primary reviewer (first author, LA) and then confirmed by the last author, NL, to ensure the continuity and precision of the process. The results of this rigorous selection process are summarized in Figure 1 and Figure 2. This updated approach also serves to make the criteria for the inclusion and exclusion of studies more explicit and thus enhance the credibility and replicability of the review methodology. In this manner, the reasons for document selection are clearly stated and supported, thereby enhancing the reliability and validity of the identified evidence.

### 2.4. Stage 4: Data Extraction

Methods in Pawson et al.’s [37,38] realist review did not require a thorough literature search or double reviews and data extraction. Thus, the literature searches were conducted by the first author, LA, peer-reviewed, and checked by the last author, NL. Final synthesis included 13 RCT-focused and 31 innovative-method sources (Figure 1 and Figure 2). The purpose of the data extraction process was to gather the framework with evidence for evaluation [37,38,43]. Thus, once the articles were extracted and found relevant to the purpose of the review, two tables were created amalgamating the data extracted from all sources [37,38,43] (Figure 1 and Figure 2). The data extraction process was validated by the last author, NL.

The accuracy and consistency of our data extraction process was validated using a multi-step approach in our study. The primary reviewer, LA, first extracted data from all included documents and coded any missing key information as not reported. Then, last author, NL, independently reviewed data, and any discrepancies were identified. After identifying the discrepancies, they were resolved through joint discussion and consensus meetings between the reviewers. This collaborative resolution process not only guaranteed that all key variables were consistently captured but also the reliability of the entire data extraction process. We undertook these steps to enhance the credibility of our findings and significantly strengthen the reproducibility of our data extraction methods through a rigorous validation process.

### 2.5. Stage 5: Data Synthesis

Consistent with the Pawson’s criteria [37,38,39,43], appraisal of the documents was made on the following two relevance and rigor criteria: (1) if the document contributed to theory building and/or testing and (2) if the method used to generate a particular document was credible and trustworthy and if the research supported the conclusions drawn from it. The data from the studies were extracted, including information regarding method of evaluation, source, and critique on the method by using criteria for appraisal of the document i.e., relevance or rigor (see Table 1). Peer-reviewed methodological papers and reviews were weighted more heavily for their theoretical and empirical rigor, while grey-literature sources (e.g., policy portals, government reports, or practice blogs) were included selectively when they offered unique contextual, or implementation insights not captured in formal publications. Relevance was determined by the extent to which a source informed theory refinement, methodological innovation, or contextual understanding. Appraisal was led by the first author, LA, and independently reviewed by last author, NL.

**Table 1:** Included sources and appraisal of methods (RCTs and innovative/accelerated designs).

Randomized Controlled Trials			
Included Sources: Peer-Reviewed Papers			
Articles	Type	Conclusion	Criteria for Appraisal of Document
1. Are community interventions evaluated appropriately? A review of six journals? [53] 1997, Canada	Systemic review of community intervention studies from six community health journals.	Many community health interventions are not evaluated properly by RCTs as they lack methodologic rigor in community health.	Rigor/relevant
2. Understanding and misunderstanding randomized controlled trials [25] 2018, USA	Methodological critique on RCTs	RCTs could play a role in building scientific knowledge to discover not what works, but why things work	Rigor/relevant
3. Randomized controlled trials: Often flawed, mostly useless, clearly indispensable: A commentary on Deaton and Cartwright [54] 2018, USA	Commentary on flaws of methodological design of RCTs	Social interventions may be among the most difficult and controversial to study with RCTs.	Rigor/relevant
4. Challenging issues in randomized control trials [55] 2010, Australia and New Zealand	Many community health interventions are not evaluated properly by RCTs as they lack methodologic rigor in community health.	Community health interventions are not evaluated properly by RCTs as they lack methodologic rigor for community health interventions.	Rigor/relevant
5. The tribulations of trials: A commentary on Deaton and Cartwright [56] 2018, USA	Commentary on methodological design of RCTs		Rigor/relevant
6. Internal and external validity of cluster randomized trials: Systematic review of recent trials [57] 2008, England	Systemic review	External validity seems poorly addressed in many trials, yet is arguably as important as internal validity in judging quality as a basis for healthcare intervention	Rigor/relevant
7. Randomized controlled trials of socially complex nursing interventions: Creating bias and unreliability? [58] 2004, England	Systemic review	Intervention bias is potentially a problem in randomized controlled trials.	Rigor/relevant
8. The randomized controlled trial: Gold standard, or merely standard? [26] 2005, USA	Book chapter: Methodological critique on RCTs		Rigor/relevant
9. Why all randomized controlled trials produce biased results [59] 2018, England	Methodological critique		Rigor/relevant
Included Sources: Descriptive Papers and Grey Literature			
Documents	Type	Source	Criteria for appraisal of document
10. The limitations of randomized controlled trials [7] 2016, USA VoxEU.org	Descriptive paper	VOX CEPR policy portal website	Relevant
11. The pitfalls of randomized control trials [28] 2010, USA	Descriptive paper	Methodological critique on American Psychological Association website https://www.apa.org (accessed on 22 January 2022)	Relevant
12. The limitations of randomized controlled trials [60] 2012, China http://aiocm.org/ (accessed on 22 January 2022)	Descriptive paper	Methodological critique at Association of integrative oncology and Chinese medicine website	Relevant
13. Are randomized control trials bad for children? [61] 2017, Italy https://blogs.unicef.org (accessed on 22 January 2022)	Blog on methodological critique at UNICEF evidence for action website		Relevant
Innovative Trials			
Peer-Reviewed Papers			
Articles		Innovative Trial Type	Criteria for appraisal of document
1. Mobile health technology evaluation: The mhealth evidence workshop [62] 2013, USA		Continuous evaluation of evolving behavioral intervention technologies (CEEBIT)	Rigor/relevant
2. Beyond the randomized controlled trial: A Review of alternatives in mhealth clinical trial methods 2016, Canada [63]		Continuous evaluation of evolving behavioral intervention technologies (CEEBIT)	Rigor/relevant
3. From innovation to impact at scale: Lessons learned from a cluster of research–community partnerships [64] 2017, USA		The IDEAS method	Rigor/relevant
4. Effect of the attachment and child health parent training program on parent–child interaction quality and child development [65] 2020, Canada			
5. Attachment and child health (ATTACH) pilot trials: Effect of parental reflective function intervention for families affected by toxic stress [66] 2020, Canada			
6. The ATTACH™ program and immune cell gene expression profiles in mothers and children: A pilot randomized controlled trial [67] 2021, Canada			
7. Building health behavior models to guide the development of just-in-time adaptive interventions: A pragmatic framework [68] 2015, USA		Just-in-time adaptive interventions (JITAIs)	Rigor/relevant
8. Just-in-time adaptive interventions (JITAIs) in mobile health: key components and design principles for ongoing health behavior support [69] 2017, USA		Just-in-time adaptive interventions (JITAIs) or micro-trials	Rigor/relevant
9. Novel methods and technologies for 21st-century clinical trials: A review [70] 2015, USA		Novel methods and technologies for 21st-century clinical trials	Rigor/relevant
10. Innovative clinical trial design for pediatric therapeutics [71] 2015, USA		Novel methods and technologies for 21st-century clinical trials	Rigor/relevant
11. Limitations of randomized controlled trial in evaluating population-based health interventions [32] 2007, Australia		Pragmatic trials	Rigor/relevant
12. Real-world evidence: How pragmatic are randomized controlled trials labeled as pragmatic? [72] 2018, Madrid		Pragmatic trials	Rigor/relevant
13. Practical clinical trials: Increasing the value of clinical research for decision making in clinical and health policy [73] 2003, USA		Practical or pragmatic trials	Rigor/relevant
14. Improving the reporting of pragmatic trials: an extension of the CONSORT statement [74] 2008, England		Pragmatic	Rigor/relevant
15. Against pragmatism: on efficacy, effectiveness and the real world [75] 2009, USA		Pragmatic	Rigor/relevant
16. The promise and pitfalls of pragmatic clinical trials for improving health care quality [76] 2018, USA		Pragmatic trials	Rigor/relevant
17. Ethical issues in pragmatic randomized controlled trials: a review of the recent literature identifies gaps in ethical argumentation [77] 2018, Canada		Pragmatic trials	Rigor/relevant
18. Making trials matter: pragmatic and explanatory trials and the problem of applicability [78] 2009, England		Pragmatic	Rigor/relevant
19. ‘Pragmatic’ and ‘explanatory’ attitudes to randomized trials [79] 2017, England		Pragmatic	Rigor/relevant
20. A pragmatic view on pragmatic trials [80] 2011, USA		Pragmatic	Rigor/relevant
21. Realist RCTs of complex interventions—An oxymoron [81] 2015, England		Realist RCT	Rigor/relevant
22. Realist randomized controlled trials: A new approach to evaluating complex public health interventions [82] 2012, England		Realist RCT	Rigor/relevant
23. A “SMART” design for building individualized treatment sequences [83] 2012, USA		Sequential multiple assignment randomized trial (SMART)	Rigor/relevant
24. The multiphase optimization strategy (MOST) and the sequential multiple assignment randomized trial (SMART): New methods for more potent ehealth interventions [84] 2007, USA		Sequential multiple assignment randomized trial (SMART) and multiphase optimization strategy (MOST)	Rigor/relevant
25. Whole systems research methods in health care: A scoping review [85] 2019, Canada		Whole systems research (WSR) methods	Rigor/relevant
Included Sources: Descriptive Papers and Grey Literature			
Documents		Source	Rigor
26. FAST: Fast-fail trials [86] 2015, USA https://www.nimh.nih.gov/index.shtml (accessed on 22 January 2022)		Fast-fail trial	Rigor/relevant
27. Funding agency seeks success in fast-fail clinical trials [87] 2012, USA https://www.spectrumnews.org/news (accessed on 22 January 2022)		Fast-fail trial	Relevant
28. From best practices to breakthrough impacts: A science-based approach to building a more promising future for young children and families [88] 2016, USA https://developingchild.harvard.edu/ (accessed on 22 January 2022)		The IDEAS method	Rigor/relevant
29. Centre of developing child at Harvard University website USA https://developingchild.harvard.edu/our-approach/partnerships-engagements/frontiers-of-innovation/ (accessed on 22 January 2022)		The IDEAS method	Relevant
30. Just-in-time adaptive interventions (JITAIs) are not self-help [89] 2018, England https://blogs.ucl.ac.uk/ (accessed on 22 January 2022)		Just-in-time adaptive interventions (JITAIs)	Relevant
31. Using rapid-cycle research to reach goals: Awareness, assessment, adaptation, acceleration [90] 2015, USA https://pbrn.ahrq.gov/ (accessed on 22 January 2022)		Rapid-cycle research	Relevant

At a face-to-face meeting, we developed an extraction tool to promote a consistent approach following Pawson’s recommendations [37,38]. We conducted virtual and in-person meetings to develop and refine the extraction tool [91], and assessed the degree to which each method: (1) enabled evaluation, (2) was theory driven, (3) offered clear guidelines, (4) provided clear methods or tools (5) fostered innovation, (6) was fast, (7) was generalizable, (8) works for who and under what circumstances, and (9) focused on children and child-related research. Based on the extraction tool, we challenged and criticized the emerging findings from the above literature searches (Figure 1 and Figure 2) [91]. We developed a connection across the extracted sources based on the contents of the extraction tool [37,38,43,91]. Finally, we explicitly linked all sources to the contents of extraction tool and created a new table (Table 2) based on the data extracted from Table 1.

## 3. Results

The identified sources that described or criticized the RCT method and innovative trial methods, are presented in Table 1. We systematically recorded the number of documents that met our inclusion criteria to ensure a balanced and comprehensive evaluation. For the RCT review, our searches and subsequent screening resulted in the inclusion of 13 documents, while for innovative trial methods, 31 documents were ultimately included in the final analysis. These documents were further distributed across several subcategories to capture the breadth of innovative approaches: for instance, categories such as continuous evaluation of evolving behavioral intervention technologies (CEEBIT) [94], fast-fail trials [87], the IDEAS method [106], just-in-time adaptive interventions (JITAIs) [69,89,95,96], novel methods and technologies for 21st-century clinical trials [70], pragmatic trials [74,78,80], rapid-cycle research methods [90], realist RCTs [81], SMART and MOST methods [84,104], and whole systems research (WSR) methods [85] were all represented with specific counts (see Table 1). It is essential to understand each of the new, accelerated methods before moving to comparisons among the methods. Thus, we described the innovative methods individually and compared them with RCTs. Also, the contents of the extraction tool that we developed in Stage 5 served as a synthesis of findings that compared all methods, and how they link to children and child development research. Thus, we described the criteria for evaluation of each innovative method and RCTs to make recommendations for early childhood intervention evaluation in Table 2.

In addition to outlining the core features of each intervention method, our analysis also identified key strengths and limitations. For example, RCTs are considered to have robust internal validity due to strict randomization and control procedures; however, they are criticized for being rigid, expensive, and applicable to limited real-world conditions [55]. In contrast, newer methods such as pragmatic trials are noted for their external validity and the ability to generalize findings to heterogeneous populations, but they may compromise on the level of control and the accuracy of measuring intervention effects [75]. Based on feasibility, adaptability, and the overall balance between internal and external validity, all methods are further evaluated, offering a more nuanced comparison of their potential and limitations.

### 3.1. Continuous Evaluation of Evolving Behavioral Intervention Technologies (CEEBIT)

Mohr and colleagues [94] proposed the CEEBIT for the evaluation of mobile health (mHealth) applications or apps designed to address health problems by using Behavioral intervention technologies (BITs). BITs are online, mobile interventions designed to support patients in altering health behaviors. The CEEBIT method integrates data from multiple BITs provided by one organizational system to achieve clinical outcome. To elaborate, CEEBIT uses data from BITs, analyses them to identify inferior BITs, allocates patients to BITs, and eliminates inferior BITs from the system [94]. According to the method developers, the CEEBIT methodology may have improved computing power to continuously evaluate app efficacy throughout trial duration and account for changing app versions through a sophisticated elimination process. CEEBIT thus allows for rapid iteration of intervention programs, randomization methods, and even participant inclusion and exclusion criteria in response to emerging information about intervention effectiveness.

In contrast to RCTs, this method enables researchers to account for app (intervention) versions, changing eligibility criteria, and changing group assignments. The traditional RCTs could take up to 17 years from initial research to full implementation which is fundamentally incompatible with the behavioral intervention technology environment, where technology advancement and changes in consumer expectations happen quickly, necessitating rapidly evolving interventions [94]. Additionally, we could not find enough literature to support that this method requires a theory of change. Overall, CEEBIT method allows real-time adaptation of digital health interventions, reducing lag in intervention refinement, however, this method has limited applicability to non-digital interventions that may introduce bias if not well-controlled [94].

### 3.2. Fast-Fail Trials

Fast-fail trials test and analyze innovative interventions and their clinical targets especially for treating mental health illnesses [86]. The fast-fail method aims to tie innovative interventions to underlying biological disease mechanisms from the very beginning [86]. Interventions that fail to improve the target outcomes are discarded in the initial trial stages, saving valuable resources to test other promising interventions. Unlike RCTs, this method allows faster iterative mid-course adaptations. Also, we did not find enough evidence to support that this method requires a theory of change. Fast-fail trials efficiently eliminates ineffective interventions, saving time and resources, however, this method may prematurely discard interventions with delayed effects, making it less suitable for long-term child development studies [86].

### 3.3. IDEAS Method

The Innovate, Develop, Evaluate, Adapt, and Scale (IDEAS) method involves flexible approaches to facilitate intervention evaluation, and fast-cycle iteration by utilizing research methods and tools in specific and rigorous ways [64]. The IDEAS was designed to foster a rapid generation of evidence to improve the health of children facing the consequences of early life adversities [88,107]. The IDEAS method consists of three main components: (1) Theory of change, or detailed set of beliefs (strategies, intervention targets, outcomes, and moderators) about identifiable changes expected from an intervention [64]. (2) Program and materials development involves creating high quality materials that includes those used to train staff, and implementation checklists and fidelity scales [88]. (3) Evaluation plan includes measures and processes that are used to evaluate the impact of an intervention [88].

The IDEAS method is guided by four principles including precision, fast-cycle iteration, co-creation and shared learning [64,88]. The guiding principles increase the likelihood that study results are relevant to real world contexts with feasibility at scale [88,108,109]. A typical research design includes a planning and development stage, feasibility study, early-stage pilot study, later stage study and evaluation. Overall, this method requires researchers to employ precision of measurable intervention targets, moderators, and outcomes to test the intervention in a faster and cost-effective manner. Like RCTs, the IDEAS method requires strict protocols and use of highly specific measures to maximize internal validity, which has been used to test childhood interventions [64]. Unlike RCTs, the IDEAS method allows mid-course adaptations to be embedded as a part of research design, and the results may be more readily generalized to a wider selection of participants, maximizing external validity. Overall, IDEAS method incorporates co-creation and shared learning, enhancing applicability to real-world settings. However, it requires extensive stakeholder collaboration, which may not always be feasible.

### 3.4. Just-in-Time Adaptive Interventions (JITAIs) or Micro Trials

JITAI is the intervention design that alters the provision of an intervention o best suit the recipient’s changing status and context to provide the right intervention components at the right times and locations to optimally support individuals’ health behaviors [68]. Modern mobile and sensing technologies allow real-time observations of an individual’s internal state and the context to administer JITASIs flexibly in terms of time and location [68,96]. JITAIs have become increasingly popular to support behavioral changes in the domains of physical activity [110], alcohol consumption [100], mental health [111], smoking [112], and obesity [113]. When designing JITAIs, four components need to be considered [114]. First, a decision point is a time at which an intervention decision is made (e.g., following each random prompt for self-report) [111]. Second, intervention options are a variety of possible actions that may be taken at any given decision point. For example, seeking support, sources of support (e.g., smartphone app), or media employed to administer support (e.g., phone calls, text messaging) [113]. Third, the tailoring variable is information related to the individual that is used to decide when and which intervention to provide. Finally, decision rules operationalize the adaptation by specifying which intervention option to offer, for whom, and when [115].

In contrast to RCTs, the motivation for the JITAI approach is underpinned by the idea that timing plays a significant role in exploring whether support provision will be beneficial. The right time to administer JITAI is determined by a theory of change, namely how and why the desired change is expected to unfold over time in a particular context. Also, JITAIs can be adapted to modify the type, amount, and timing of support resulting in more personalized health care. Despite JITAIs’ increasing use and appeal, research on their development and evaluation is in its initial stages. Many JITAIs have been developed with little empirical evidence, theory, or accepted treatment guidelines [116]. Taken together, JITAIs provide timely interventions tailored to individual needs, increasing effectiveness, however, this method requires complex real-time data collection and analytics, which may not be available in all contexts.

### 3.5. Novel Methods and Technologies for 21st-Century Clinical Trials

Novel methods and technologies for 21st-century clinical trials has been described in the literature to include five approaches namely disease modeling and simulation, alternative trial designs, novel objective outcome measures, virtual research visits, and engagement of research participants to combat the cost and time required to determine whether novel interventions are safe and efficacious [70]. Unlike RCTs, these novel methods address specific limitations of RCTs and do not yet comprise stand-alone methodologies.

In disease modeling and simulation, disease progression may be better estimated as well as the trajectory of disease and factors that contribute to variance [117]. Alternative trial designs may help identifying maximum tolerated dosage. This method also encourages the researchers to retain participants already enrolled in a single trial for Phases 1 and 2 instead of recruiting new participants in Phase 3 trials to maximize efficiency [118]. The novel outcome measures approach may be used to augment evaluation measures [119]. Virtual visits may occur by telephone, video, or asynchronous communication platforms, such as texting, which may facilitate greater participation, decrease burden on the participants, and decrease variability in assessments, potentially increasing generalizability to a variety to contexts [70]. These methods also typically involve research participants in advisory committees and providing them with an unprecedented voice in research [120]. The approach incorporates several new innovations, like disease modeling, alternative trial designs, and virtual research visits, but these components have not been consolidated into a single, distinct, standalone methodology [70,120]. Although developed to address cost and time limitations, these methods have been used exclusively in drug trials, not conducive to community-based or non-pharmaceutical interventions and may not capture all the critical contextual complexities that influence real world intervention effectiveness.

### 3.6. Pragmatic (or Naturalistic or Practical) Trials

Pragmatic trials are designed to test interventions with flexible eligibility criteria to maximize effectiveness of an intervention and generalizability of results [72]. In contrast, pragmatic trials are overlaid on RCTs and designed with strict eligibility criteria to maximize an intervention’s efficacy in controlled settings [74,78,79]. In order to design a pragmatic trial, a pragmatic-explanatory continuum indicator summary (PRECIS) tool is used [121]. This tool has ten dimensions related to intervention design presented on a graphical, ten-spoked wheel diagram rated from one (most explanatory) to five (most pragmatic) [78,121]. PRECIS scores range from 0 to 50; scores from 0 to 15 suggest an explanatory trial (trending toward traditional RCT design); scores from36 to 50 suggest a pragmatic trial; and scores from 16 and 35 suggest an interim where trial design balances pragmatic and explanatory domains [122]. The PRECIS tool has broad application; however, it has not been validated. A revised version called PRECIS-2 has been developed scored on a five-point Likert continuum from one (very explanatory) to five (very pragmatic) [123]. PRECIS -2 exhibits good inter-rater reliability with seven out of nine domains having an intra-class correlation coefficient over 0.65 [123].

Unlike RCTs, pragmatic trials may be undertaken in real-life settings to achieve maximum external validity; thus, the approach has gained the interest of policy makers [76,121,123]. Blinding in pragmatic trials is practically challenging as broad patient populations are targeted and are typically embedded in standard healthcare settings [74]. Like RCTs, pragmatic trials do not require the development of an explicit theory of change in research design. Taken together, a researcher using public money may opt to design a pragmatic trial if results are likely to produce evidence for majority of people suffering with a medical condition in multiple heterogeneous contexts [72,75,80]. In short, pragmatic trials focus on effectiveness in real-world conditions rather than idealized clinical settings, however, this method may suffer from heterogeneity in intervention effects and lack of strict control mechanisms [80].

### 3.7. Rapid-Cycle Research Method

The rapid-cycle research method employs approaches to accelerate the research-to-action cycle [90] within a timeframe of short duration (i.e., a month or less) and is comprised of multiple cycles of minor amendments in the initial phases of intervention testing [124]. This method has been used to assess care improvement processes [125]. The rapid-cycle research method consists of six phases [90]. In the preparation phase, researchers identify partner organizations who may have experienced a similar health issue or problem. In the problem exploration phase, the clients’ perspectives are explored to gain a deeper understanding of the issue. In the knowledge exploration phase, a problem is explored from a distinct perspective, e.g., if and how other organizations or providers have overseen it. The solution development phase involves the identification of the most specific possible intervention to address the issue. The solution testing phase involves evaluating the identified intervention using quantitative methods, including pilot studies, pragmatic, or explanatory trials, and/or qualitative methods. Lastly, the implementation/dissemination phase involves scaling the efforts once the intervention is effective [90]. Unlike RCTs, this method provides thorough strategies to test interventions in interdisciplinary settings via rapid iterative processes. Overall, rapid-cycle research method enables fast-paced evaluation and iterative improvements, however, this method may lack methodological rigor compared to RCTs and the findings may not always be generalizable [90].

### 3.8. Realist RCTs

Realist RCTs have been proposed to address limitations of traditional RCTs in understanding what works for whom in multiple contexts and in underlying processes of implementation, while preserving internal validity in estimating effects [82]. Consistent with realist evaluation principles, realist RCTs proceed in three stages [101]:

- Stage 1 involves developing a priori context, mechanism, and outcome (CMO) hypotheses and elaborating a theory of change via a diagrammatic logic model.
- Stage 2 involves refining or augmenting CMO hypotheses before collecting quantitative, follow-up data.
- Stage 3 involves evaluating the CMO hypotheses using process and outcome data.

These phases enable researchers to evaluate the effectiveness of an intervention, as well as what interventions work best for what population and in which settings [101]. Unlike RCTs, realist RCTs are more focused on understanding and empirically analyzing when and how an intervention works in the different contexts in which it is implemented. They also examine how intervention may differ for various groups by anticipating the diversity of intervention mechanisms, depicting this in a theory of change, and then presenting it empirically [82,101,126]. There has only been a theoretical description of realist RCTs [82], and only one example of implementation was identified [101]. In short, the realist RCTs integrate contextual analysis, making it more applicable for community-based interventions. However, they have limited real-world applications and carry a higher risk of subjective interpretation of findings [82,101].

### 3.9. Sequential Multiple Assignment Randomized Trial (SMART) and Multiphase Optimization Strategy (MOST) Methods Used in e-Health

Both SMART and MOST methods are used to evaluate electronically delivered interventions via e-Health [64]. SMART is an innovative research design especially suited for building time-varying adaptive interventions [83] in which everyone may be randomly assigned to conditions several times. Developing an adaptive intervention strategy requires addressing questions such as: What is the best sequencing of intervention components? Which tailoring variables could be employed? How sporadically and at what times should tailoring variables be reassessed and an opportunity for changing the amount and/or type of intervention be displayed? Is it better to provide treatments to individuals on a regular schedule or to let them pick from a menu of treatment options [103]? This approach enables researchers to evaluate interventions in a holistic yet rigorous manner, considering the sequence in which components are displayed rather than each component in isolation. In short, a SMART trial provides an empirical basis for selecting appropriate decision rules and tailoring variables with the end goal of developing evidence-based adaptive intervention strategies, which are then evaluated in a subsequent RCT.

MOST is an alternative way of developing, optimizing, and evaluating interventions [84]. It incorporates the standard RCT, but before the RCT is undertaken, MOST also uses a method for identifying what components are active in intervention and what levels of each component lead to the best outcomes [63]. MOST consists of the three phases, screening, refining, and confirming [84]. In the screening phase, intervention components are efficiently identified for inclusion in an intervention or rejection based on their performance. In the refining phase, the selected components are fine-tuned, and issues such as optimal levels of each component are investigated. In the confirming phase, the optimized intervention composed of selected components delivered at optimal levels, is evaluated in a standard randomized controlled trial.

Unlike RCT approaches of constructing an intervention a priori and post-hoc testing to determine efficacy followed by appropriate revision of intervention design, SMART and MOST methods are designed to accelerate the intervention-RCT-post hoc-analysis-revision of interventions to reduce bias [84]. Both SMART and MOST incorporate standard RCTs. While innovative, they do not qualify as accelerated methods. SMART and MOST methods identify the active components of intervention to identify best outcomes [84], but neither require a theory of change. Overall, SMART and MOST are tailored towards adaptive interventions and e-Health applications [103,127]. However, this method suffers from complexity in execution, making them challenging to implement at scale.

### 3.10. Whole Systems Research (WSR) Methods

WSR methods require acknowledging unique patient, family, community, and environmental (i.e., system) characteristics and perspectives by including a qualitative evaluation component [85]. Thus, WSR methods must involve mixed methods approaches to improve a range of relevant and holistic outcome measures of any intervention [85]. WSR are not only focused on the active ingredients of a system but also to the context of the system as a whole, as the individual components of most whole systems are inseparable, complementary and synergistic [105]. There are no clear guidelines or methods available for intervention design as WSR methods are emerging and still evolving. WSR methods must include cyclical, flexible and adaptive designs [85] which differs from RCTs that prohibit adaptability to new finding or contexts. A theory of change is not a specific formal requirement for the use of WSR methods. However, as WSR is developing as a methodology, theory of change is expected to become a default practice which will help researchers to design, implement, and evaluate whole-system interventions in context more effectively [85,105]. Overall, WSR methods are still emerging, and there are no standardized guidelines to replicate or compare across studies. WSR typically includes holistic mixed methods approaches and embrace patient, family, community and environmental factors [85]. However, this complexity may pose challenges to methodological rigor.

### 3.11. Overall Comparative Analysis of RCTs and Innovative Evaluation Methods in Child Development Research

Our findings revealed that RCTs do not allow mid-course adaptations and pose a threat to generalizability particularly in child development research (see Table 2) [7]. Instead, innovative methods are deemed faster to draw research conclusions than RCTs. Mid-course adaptations can be made in almost all the new methods. Most of the innovative methods have clear guidelines on methods, except WSR where the methods are still evolving. Among those innovative methods, the IDEAS method [64,65,66,67], pragmatic trials [97,98], and realist RCTs [82,102] deemed more suitable for child development research. The other innovative methods such as CEEBIT, fast-fail trials, JITASIs, rapid cycle research methods, SMART, MOST, novel methods, and technologies for 21st-century clinical trials, and WSR methods are relatively new methods and offer limited evidence of generalizability or applicability to evaluate early childhood-based interventions. Also, fast-fail trials and novel methods and technologies for 21st-ccentury clinical trials have only been tested in drug trials and thus, have not been used in community-based interventions. Nonetheless, these methods may be adapted to evaluate early childhood community-based interventions due to their rigor, cost, and time effectiveness; however, the evidence is currently lacking.

It should be noted that the apparent lack of child-focused applications of some newer designs (e.g., JITAIs, SMART, and MOST) may partly reflect our search strategy, which intentionally excluded child-specific terms (e.g., child development, early childhood, and pediatric) to capture methodological advances primarily reported in the adult-focused literature. As such, the results in Table 2 should be interpreted as reflecting what was captured within these search parameters rather than definitive evidence that such applications do not exist. Future reviews employing child-specific search terms may reveal additional examples of these methods applied in developmental or early-intervention contexts.

## 4. Discussion

This realist review identified and compared the methods for intervention evaluation, including traditional RCTs and newer innovative methods, for their efficiency in rapidly building knowledge to impact children’s health and development. One of the strengths of our realist review is the reviewers’ approach rooted in the social determinants of early child development [45]. The application of Hertzman’s framework served as an interpretive lens rather than an evaluative tool, guiding our assessment of whether different methodological approaches accommodate environmental, developmental, and social-contextual complexity consistent with the theory’s principles. Instead of a concrete method or formula, our review is a logic or rationale of inquiry that allows a flexible approach to explaining what works for whom in what circumstances and in what respects in child health research. Based on the criteria for evaluation of RCTs and each innovative method, as depicted in Table 2, we compared innovative methods with RCTs and each other to make future recommendations about what method may work best for whom and under what circumstances.

Our findings are in sync with Flay’s framework, which states that various methods are used for different research purposes [34]. RCTs remain the gold standard for establishing efficacy, but they may not always be the best at capturing contextual and implementation factors [55,61]. Methods such as pragmatic trials [74,78,80,97] and the IDEAS method [64] are focused more on effectiveness, thus being possibly more appropriate for community-based interventions. In other words, while effectiveness trials are conducted to determine whether interventions are successful in achieving their desired results, implementation challenges pertain to actual circumstances that affect the uptake and sustainability of an intervention in the real world. Such innovative methods as pragmatic trials and realist RCTs [81,101] act as a bridge between these two aspects by incorporating implementation aspects into the effectiveness evaluations. This is important in child health intervention research as many interventions that show promise in theory fail to translate into practice owing to implementation barriers [10,88,108]. The two concepts can be distinguished to enable researchers and practitioners to devise specific plans for improving the effectiveness and applicability of an intervention in various contexts [34].

The identified sources that criticized the RCT method demonstrated that early childhood interventions have been developed and tested to improve developmental outcomes in children by employing RCTs [3,4,128]. However, the effects have not improved over time as large numbers of young children continue to face the burden and consequences of chronic stressors [9,10,11,88]. Further, RCTs may be a method of choice to test pharmaceuticals [7,26,32], however, RCTs may be less useful in evaluating community interventions due to heterogenicity of contexts [32,53,58]. RCTs of community interventions may be challenged to examine what works without describing the underlying processes of implementation and mechanisms of action in multiple contexts [129]. The situation becomes even more challenging when testing early interventions for children as the interplay of the developing brain with the environment needs to be taken into consideration, as suggested by Hertzman’s theory [46,48,51]. Thus, many of the identified innovative methods seem to be more practical, context-based, and hold the potential to accelerate the research-to action-cycle in child and community health [72].

As Hertzman’s theory focuses on how social environments affect child development, evaluating intervention methods needs a consideration of these contextual influences [45,46,48,49,51]. Although RCTs are powerful to establish efficacy, they may lack the necessary flexibility to evaluate interventions in various community contexts [9,10,11,61,88]. Pragmatic trials [97] and realist RCTs [101] are more consistent with Hertzman’s framework because they emphasize on what works, for whom, and under what conditions. IDEAS methods that enable real-time adaptation, may be more appropriate to capture these complex interactions [64,65,66]. Furthermore, a realist review approach deemed appropriate for this study because it aligns with Hertzman’s theory by focusing on context-sensitive evidence synthesis [51]. Traditional systematic reviews differ from realist reviews in that they explore how interventions function differently across populations and settings, which is important for evaluating methods intended to promote early childhood health and development [37,130].

Compared to RCTs, innovative methods may be deemed faster to draw research conclusions and allow mid-course adaptations that facilitate fast progress through the research-to-action-cycle [64,65,66,90]. They also involve early evaluation of interventions in pilot programs or policy implementation process, thus enabling intervention adaptation, change, and iterative improvement [64,65,66,90], that may make them more suitable to test community-based interventions in diverse contexts. Among those innovative methods, CEEBIT, JITASIs, SMART, and MOST have been employed in behavioral and psychological research to test weight management, mental illness, and physical activity interventions [95,104,127]. The rapid cycle research method have been used in behavioral and addiction research [90,99,100]. Fast-fail trials have been used to test the effectiveness of psychiatric medications [131], whereas novel methods for 21st century trials have only been used in pharmaceutical trials to date. However, this method can be adapted to evaluate community-based interventions due to their rigor, cost, and time effectiveness features [72,82]. Also, WSR methods have been employed to test ayurvedic, chiropractic, homeopathic, and midwifery intervention [85]. As such, CEEBIT, JITASIs, SMART, MOST, and WSR methods, novel methods, technologies for 21st-century clinical trials, and fast-fail trials have not been employed to test community or early interventions to date.

Theory is a cornerstone of the realist approach that explains how and why interventions work by uncovering the underlying generative mechanisms through context–mechanism–outcome configurations [132]. It emphasizes that theories are not fixed but are continually refined through an iterative process of testing against emerging evidence [37,38]. While some innovative methods, such as pragmatic trials [80] and fast-fail trials [87], may function without formally incorporating a theory of change, integrating this theoretical lens can provide additional clarity on the underlying mechanisms and contextual factors that shape intervention outcomes. By articulating a clear theory of change, researchers can better trace the pathways through which interventions are expected to yield their effects, thereby enriching the interpretation of evidence and ensuring a more comprehensive understanding of what works, for whom, and under what circumstances [45,46,47,48,49,50,51].

In our comparative analysis, the contrast between methods that explicitly incorporate a theory of change, such as the IDEAS method [106] and realist RCTs [101], and those that do not becomes evident. Methods grounded in a theory of change tend to offer a more nuanced understanding of the complex interactions between intervention components, participants, and their social environments [47]. This theoretical foundation facilitates mid-course adaptations and helps to elucidate the contextual influences that may affect the implementation and sustainability of interventions [65,66,133]. Recognizing this, our findings suggest that even innovative methods which traditionally do not require a theory of change could benefit from its integration, thereby improving both methodological rigor and practical applicability in community-based child health interventions.

Among the innovative methods, IDEAS method, pragmatic trials, and realist RCTs have been used to evaluate community and early childhood interventions childhood interventions [64,65,66,82,97,134]. However, pragmatic trials do not allow for mid-course adaptations and may suffer limitations due to heterogeneity of intervention effects [76,77]. Moreover, a negative result in a pragmatic trial cannot provide information on whether the intervention is effective under optimal conditions [72,74,79]. In comparison, the IDEAS method provides opportunities to conduct rigorous research with fewer resources and a shorter time frame, and the iterative process enables faster replication [64,88,108]. Results may be more readily applied in real-life settings or generalizable due to the co-creation and shared learning components [64]. The IDEAS method also rivals RCTs as they result in more informed care faster, via the conduct of micro-trials for fast iteration and shared learning, while still adhering to the RCT standards of using feasibility and pilot trials [65,66,134].

To preserve internal validity, the IDEAS method and realist RCTs are better-suited methods as they require a theory of change to be included in their research design to help understand what works for whom in multiple contexts and underlying implementation processes. The realist RCTs may hold the potential to overcome some of the difficulties faced by the RCT method when considering the impact of social context and individual interpretation. Likewise, the IDEAS method provides clarity and accuracy in understanding what an intervention entails and how the intervention is helpful in achieving a high degree of precision in the development and testing process [64]. However, we did not find enough evidence to comment on the generalizability of results from the realist RCTs.

From a realist perspective, longitudinal cohort infrastructures can complement realist review findings by enabling iterative testing of CMO configurations in real time [37,38,39]. Integrated within adaptive evaluation frameworks such as IDEAS, these data systems allow continuous feedback between evidence and implementation, functioning as living laboratories for contextual refinement. For instance, South Australia’s BEBOLD platform [135] and the UK’s emerging federated data networks [136] demonstrate how linked population data can support Population Health Management through real-time quasi-experiments, guiding adaptations in policy and practice. Embedding approaches like IDEAS within these infrastructures can help close the know–do and do–know gaps, accelerating equitable, context-sensitive innovation in child-health research and implementation.

Taken together, the IDEAS method tends to preserve both internal and external validity by having the requirements of incorporating a precise theory of change, program materials, and concrete evaluation plan, as well as co-creation and shared learning, which makes it a better suitable method to test early community interventions [64,65,66,67]. While the IDEAS framework is described as particularly integrative for community-based interventions, this should not be interpreted as a hierarchical comparison; rather, IDEAS, pragmatic trials, and adaptive methods such as JITAIs operate at different levels of scope and purpose, each offering complementary strategies suited to distinct contexts and implementation needs.

In this realist review, we discussed and analyzed a framework for working with the complexity of real-life implementation in community and child development research. This approach allowed for an equal focus on what works, for who, and under what circumstances, as well as what does not work, in an attempt to learn from failures and maximize learning across policy, disciplinary and organizational boundaries [38,43]. This position aligns with recent pragmatist and complexity-informed accounts that prioritize transferability/portability over universal generalizability and advocate for situated judgement in applying evidence to real-world systems [12].

### Limitations and Future Recommendations

Despite the increasing use of realist review methods, they may be limited due to the quality assessment of included source documents. Source documents are included if they are judged to contribute to theory building and/or testing and be credible and trustworthy, as study conclusions are drawn logically from research conducted [137,138]. This approach contrasts with other systematic review methods that typically require complex quality assessment considering the risk of bias [139]. The pragmatic approaches of realist reviews focus on what works for whom in what circumstances, in what respects, and how. This approach to quality assessment was thus consistent with our review aims. Moreover, in our initial search strategy, we excluded child development specific terms and only sought broad intervention evaluation methods, if many of the methods developed for adult populations could be adapted for child health settings. However, given the significance of contextual factors on child development, we recognize that incorporating specific terms, such as child development, early childhood, infant, and pediatric might have improved the search results and thus the capture of relevant literature. Future reviews could consider incorporating these child development related terms. Additionally, this review did not include formal patient and public involvement in shaping the question, screening, and interpreting evidence, or drafting recommendations; our outreach focused on methodological experts rather than patient and public voices. This is a limitation to be addressed in future work to reinforce relevance and equity.

After comparing the characteristics, strengths, and weaknesses of RCTs against the newer, accelerated methods, the following recommendations may be made:

1. The IDEAS method may provide better, faster, more organized, and flexible approaches to facilitate intervention evaluation for community-based child health interventions than the traditional RCTs and other innovative or accelerated methods.
2. The IDEAS method may combat issues of internal and external validity of other innovative and accelerated methods. This method enables research teams to test the effectiveness of interventions in different contexts because of the flexibility and short duration of micro-trials.
3. The IDEAS method may allow more effective and rapidly scalable, yet personalized interventions for children vulnerable to less-than-optimal health and development in the community than either RCTs or innovative and accelerated methods.
4. An evidence base for applying the IDEAS method has increased [64], but more evidence of the effectiveness of the method is nonetheless required.

However, it is crucial to note that the evidence for employing the IDEAS method to test the effect of early interventions on child development is growing [65,66,134]. Continued application and reporting of IDEAS-based evaluations will be essential for further validation in diverse populations and service contexts. Despite the need for ongoing testing, the significant advantages of the IDEAS method (its adaptability, co-creation with stakeholders, and efficiency) make it a promising approach for evaluating early interventions (Table 2). By contrasting traditional efficacy-focused RCTs with newer iterative and context-responsive frameworks, this review highlights a critical shift in child-health implementation science, from testing interventions in isolation to refining them dynamically within real-world systems. Applying frameworks like IDEAS enables faster learning, stronger stakeholder engagement, and greater adaptability to the complex realities of family and community contexts.

While our realist review focuses on evaluation methods (e.g., realist RCTs, IDEAS), recent implementation science advances, such as co-production, learning health systems, and embedded implementation trials, blur the line between evaluation and knowledge mobilization and complement these methods by linking evidence generation with implementation and practice-based learning in real time [12]. Although several approaches, including IDEAS, have already been tested in child populations (see Table 2), part of the methodological evidence base still comes from adult settings; portability should not be assumed across all contexts, and further child-specific evaluations remain warranted.

This perspective aligns our findings with a long line of pragmatic and practice-based scholarship, from Lind’s empirically grounded problem-solving and Dewey’s experiential pragmatism to engaged scholarship, theory-in-practice [40], and modern knowledge mobilization and embedded evaluation movements [37,38,39,42,43]; each seeking to bridge evidence and action through iterative, relational approaches that emphasize transferability and contextual learning over generalizability. These traditions collectively reinforce the realist logic underlying this review: to understand not merely whether interventions work, but how, for whom, and under what circumstances.

## 5. Conclusions

This realist review compared the traditional RCTs with newer adaptive designs for evaluating community-based child-health interventions. Although RCTs provide strong internal validity, their rigidity, cost, and long timelines often limit their suitability for early, real-world contexts. Growing evidence supports pragmatic trials, realist RCTs, and especially the IDEAS framework (Innovate, Develop, Evaluate, Adapt, Scale) as context-sensitive, theory-driven, and co-created approaches that enable rapid-cycle iteration while preserving rigor. Grounded in Hertzman’s bio-psycho-social perspective, these methods address what works, for whom, and under what circumstances.

The IDEAS method offers a practical and comprehensive pathway for bridging scientific rigor with real-world relevance. It facilitates personalized, scalable, and cost-effective approaches to child-health promotion. Collectively, the findings of this review signal a needed paradigm shift in implementation science—from more trials to better-designed trials that close both the know-do and do-know gaps. By integrating evidence generation with adaptation and stakeholder feedback, frameworks such as IDEAS can accelerate translation, enhance fidelity and sustainability across settings, and ultimately advance equitable outcomes for children and families.

## Figures and Tables

**Table 2:** Summary matrix of methods against realist-informed criteria.

Method	Evaluation	Theory Driven	Clear Guidelines	Mid-Course Adaptations	Allows Innovation	Fast	Generalizability	Works for Who and Under What Circumstances	Focused on Children/Child Development
Traditional Randomized controlled trials (RCTs)									
RCTs	**✓**	Not enough evidence to comment	**✓**	Not enough evidence to comment	Not enough evidence to comment	Not enough evidence to comment	Not enough evidence to comment	Broadly applicable	**✓** e.g., [92,93]
Innovative Methods									
Continuous Evaluation of Evolving Behavioral Intervention Technologies (CEEBIT)	**✓**	Not enough evidence to comment	**✓**	**✓**	**✓**	**✓**	Not enough evidence to comment	Behavioral and psychological research [94]	Not enough evidence to comment
Fast-Fail Trials	**✓**	Not enough evidence to comment	**✓**	**✓**	**✓**	**✓**	Not enough evidence to comment	Psychiatric clinical trials [87]	Not enough evidence to comment
The IDEAS Method	**✓**	**✓**	**✓**	**✓**	**✓**	**✓**	**✓**	Early childhood interventions [64,65,66,67]	**✓** [64,65,66,67]
Just-in-Time Adaptive Interventions (JITAIs) or Micro Trials	**✓**	Not enough evidence to comment	**✓**	**✓**	**✓**	**✓**	Not enough evidence to comment	Behavioral and psychological interventions related to weight management and physical activity [68,69,89,95,96]	Not enough evidence to comment
Novel Methods and Technologies for 21st-Century Clinical Trials	**✓**	Not enough evidence to comment	**✓**	**✓**	**✓**	**✓**	Not enough evidence to comment	Pharmaceutical trials [70]	Not enough evidence to comment
Pragmatic Trials	**✓**	Not enough evidence to comment	**✓**	Not enough evidence to comment	**✓**	**✓**	**✓**	Broadly applicable	**✓** [97,98]
Rapid-Cycle Research Method	**✓**	Not enough evidence to comment	**✓**	**✓**	**✓**	**✓**	Not enough evidence to comment	Behavioral and addiction research [90,99,100]	Not enough evidence to comment
Realist RCTs	**✓**	**✓**	**✓**	**✓**	**✓**	**✓**	Not enough evidence to comment	Early childhood interventions [101]	**✓** [82,102]
Sequential Multiple Assignment Randomized Trial (SMART) and Multiphase Optimization Strategy (MOST)	**✓**	**✓**	**✓**	**✓**	**✓**	**✓**	Not enough evidence to comment	Behavioral and psychological interventions related to weight management, mental illness, and physical activity [84,103,104]	Not enough evidence to comment
Whole Systems Research (WSR) Methods	Not enough evidence To comment	Not enough evidence To comment	**✓**	**✓**	**✓**	**✓**	Not enough evidence To comment	Ayurvedic, chiropractic, homeopathic, and midwifery interventions [85,105]	Not enough evidence to comment

## Data Availability

The data supporting the findings of this study are derived from published and grey literature sources referenced in the manuscript and are available within the article. Further inquiries can be directed to the corresponding author.
